# Growth of poorly differentiated endometrial carcinoma is inhibited by combined action of medroxyprogesterone acetate and the Ras inhibitor Salirasib

**DOI:** 10.18632/oncotarget.867

**Published:** 2013-02-27

**Authors:** Raya Faigenbaum, Roni Haklai, Gilad Ben-Baruch, Yoel Kloog

**Affiliations:** ^1^ Department of Neurobiology, The George S. Wise Faculty of Life Sciences, Tel Aviv University, Tel Aviv, Israel; ^2^ Department of Obstetrics and Gynecology, Sheba Medical Center, Tel Hashomer, Sackler School of Medicine,Tel Aviv University, Tel Aviv, Israel

**Keywords:** Endometrial cancer, medroxyprogesterone acetate, FTS, Ras, ERα, ERβ

## Abstract

Type 2 endometrial carcinoma (EC) is a poorly differentiated EC. Unlike type 1 EC, which responds to hormonal treatment (progestins), type 2 EC is refractory to hormonal treatment because of its low expression of active estrogen and progesterone receptors (ER, PR). The aim of this study was to develop a novel drug combination designed to treat these aggressive type 2 EC tumors without surgery and with fertility potential preserved. We examined the effects of combined treatment with the progestin medroxyprogesterone acetate (MPA) and the Ras inhibitor S-farnesylthiosalicylic acid (FTS; Salirasib). Because FTS can induce cell differentiation in tumor cells, we examined whether FTS could induce re-differentiation of type 2 EC cells, thereby sensitizing them to MPA. We found that FTS reduced Ras-GTP, phospho-Akt, and phospho-ERK, and that these reductions all correlated with a decrease in ERα phosphorylation. Combined treatment with FTS and MPA induced stronger reduction in USPC1 type 2 EC cell numbers than the reduction induced by either drug alone. MPA caused ERα degradation. Death of the cells was caused by MPA but not by FTS. The phosphorylated ERα induces gene transcription manifested by enhanced cell proliferation and survival. The combination of FTS and MPA, by reducing the mRNA expression of ERα-mediated genes (i.e. *PR, c-fos* and *ps2*/*TFF1*), inhibited tumor growth and enhanced the death of type 2 EC cells. These promising results might herald a novel treatment for the highly aggressive, incurable type 2 endometrial carcinoma.

## INTRODUCTION

The endometrium undergoes cyclic regeneration in response to ovarian steroid hormones. Proliferation of endometrial cells is induced by estrogen and inhibited by progesterone [1]. Endometrial carcinoma (EC) is the most common gynecological cancer in the Western world. Each year, EC develops in about 142,000 women worldwide, with an estimated mortality of 29% [2]. Major symptoms include dysfunctional uterine bleeding, hypermenorrhea, irregular menstruation, and sterility [3]. Affected women are usually postmenopausal, but 25% are premenopausal and about 5% are younger than 40 [4, 5]. There are two types of EC. Type 1 EC, the more common type, is characterized by low-grade tumors that are related to high estrogen levels resulting from unopposed estrogen treatment, obesity [6], and history of tamoxifen use [7]. Type 1 is characterized by well-differentiated tumors, expression of active estrogen and progesterone receptors (ER, PR), and younger age of onset [4, 5]. Type 1 EC can therefore be treated in various clinical situations by progestational agents (synthetic progesterone, i.e. progestins), such as Provera (medroxyprogesterone acetate; MPA), which inhibit proliferation of endometrial glandular epithelial cells [8, 9]. Complete response to the treatment can lead to cure of the tumor without surgery and with fertility potential preserved [10]. Type 2 EC represents fewer than 10% of EC cases but accounts for more than 50% of EC-related relapses and deaths [11]. Type 2 occurs at an older age, arises from endometrial atrophy, and is not related to abnormal estrogen effects on the endometrium. These tumors are characterized by the absence or poor expression of active ERs and PRs and by high-grade histology, and are often metastatic. Thus the prognosis of type 2 EC is poor, and treatment is based mainly on surgery followed by chemotherapy and radiation [2].

Growth of the endometrium is induced by estrogen and mediated through two nuclear receptors, ERα and ERβ. Both types are transcription factors that control gene expression, which is activated either in response to ligand binding or in a ligand-independent manner [12, 13]. ERα and ERβ are products of separate genes located on different chromosomes and are differently expressed in various tissues [12, 14]. They also have opposite effects on cell proliferation and apoptosis: whereas ERα leads to cell proliferation [1, 12], ERβ modulates ERα transcriptional activity [15] and its expression increases the proteolytic degradation of ERα [16]. Progestins inhibit proliferation of EC cells by acting as ERα antagonists. They inhibit ERα action by decreasing ERα mRNA, repressing ER-related transcription of genes involved in cell growth, and activating the tumor-suppressor gene *p21* [1, 3].

Among the several genetic alterations that appear in EC is the *K-Ras* mutation which leads to constitutive activation of the K-Ras protein. This mutation occur in up to 30% of patients with type 1 EC and in 10% with type 2 EC [5, 17], and therefore Ras proteins are important targets in anti-cancer research. Activation of Ras proteins (H, N, K-Ras), which are small G-proteins, triggers a multitude of signaling cascades such as the PI3K-Akt pathway, which leads to cell survival, and the MAPK/ERK pathway, which leads to cell proliferation [18]. S-farnesylthiosalicylic acid (FTS; Salirasib) [19, 20] is a nontoxic inhibitor of all active forms of Ras proteins. Designed to mimic the farnesyl cysteine moiety of the C-terminus of Ras, it displaces active Ras from the plasma membrane and targets it for degradation [21]. FTS has been intensively studied in many types of human tumor cell lines both *in vitro* and *in vivo* [20, 22, 23] and was shown to induce autophagy in human cancer cell lines [24]. It can synergize with other anti-cancer drugs such as gemcitabine [25], 2-deoxyglucose [26], and proteasome inhibitors [27]. FTS was also shown to induce differentiation of malignant cells such as thyroid cancer cells [28] and NF1-deficient cells [29].

We aimed to develop a novel drug treatment for the aggressive type 2 EC tumors. To this end we examined the effects of combined treatment with the progestin MPA and the Ras inhibitor FTS on the growth of type 1 and type 2 EC cells (ECC1 and USPC1 cells, respectively). We tested the hypothesis that these poorly differentiated EC tumors would respond to hormonal treatment if FTS could induce their differentiation.

## RESULTS

### FTS downregulates active Ras-GTP and its downstream signaling, leading to inhibition of proliferation of ECC1 and USPC1 cells

**FIGURE 1:**
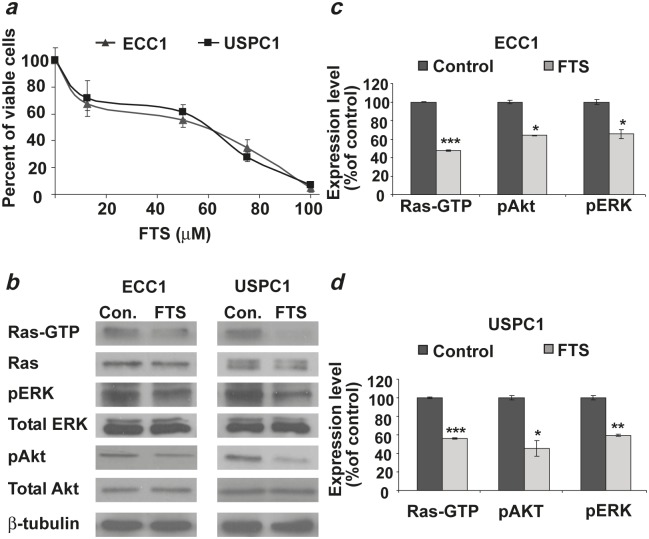
FTS downregulates active Ras-GTP and its downstream signaling, leading to inhibition of cell proliferation in ECC1 and USPC1 EC cell lines (*a*) Dose-dependent decrease in the number of viable ECC1 or USPC1 cells as a function of FTS concentration. ECC1 and USPC1 cells were plated in 24-well plates, and treated after 24 hr with 0.1% DMSO (control) or FTS (100, 75, 50, or 25 μM). After 4 days the cells were counted. The IC_50_ values of FTS in both cell lines were derived from the graph equations. (*b*) Immunoblots of Ras, Ras-GTP (active Ras), phospho-ERK, ERK, phospho-Akt, Akt, and β-tubulin (loading control) prepared from ECC1 and USPC1 control lysates and from lysates of ECC1 and USPC1 cells treated with 50μM FTS. ECC1 and USPC1 cells were plated in 10-cm plates and treated after 24 hr with 0.1% DMSO (control) or 50μM FTS. Three days later cells were lysed and subjected to western blotting with anti-pan-Ras, anti-Akt, anti-pAkt, anti-pERK, anti-ERK or anti-β-tubulin Abs (loading control). (*c*) FTS significantly decreases Ras-GTP, pERK, and pAkt both in ECC1 cells and (*d*) in USPC1 cells. There were no significant differences in total Ras, total ERK, total Akt or β-tubulin between control and FTS-treated cells. These results indicated that FTS acts in both cell lines as an inhibitor of active Ras and its downstream signaling. *, ** and *** are compared with the control for each cell line. *p < 0.05, ** *p* < 0.01, ***p < 0.001. Con, control

As shown in Figure 1*a*, we found a dose-dependent decrease in the number of viable ECC1 or USPC1 cells as a function of FTS concentration. FTS reduced the number of cells with a half-maximal (50%) inhibitory concentration (IC_50_) of 50.4 μM for ECC1 cells and 51.7 μM for USPC1 cells. Figure 1*b* shows typical immunoblots of Ras, Ras-GTP (active Ras), pERK, ERK, pAkt, Akt, and β-tubulin (loading control) prepared from lysates of ECC1 and USPC1 cells treated with 0.1% DMSO (control) or 50 μM FTS. The results of statistical analyses of these experiments are shown in Figures 1*c* and 1*d* for ECC1 and USPC1 cells, respectively. FTS treatment resulted in a significant decrease (expressed as a percentage of control cells) in Ras-GTP (ECC1: 47.4 ± 0.6%, *n* = 6, *p* < 0.001; USPC1: 56.3 ± 0.6%, *n* = 6, *p* < 0.001), pAkt (ECC1: 63.8 ± 0.3%, *p* = 0.009, *n* = 6; USPC1: 45.3 ± 8.2%, *p* = 0.01, *n* = 6), and pERK (ECC1: 65.3 ± 4.7%, *p* = 0.04, *n* = 6; USPC1: 59.5 ± 1.2%, *p* = 0.002, *n* = 6) (see Figs. 1*c* and 1*d*). There were no significant differences between the numbers of control and FTS-treated cells in lysates of total Ras, total ERK, total Akt, or β-tubulin. These results indicate that in both cell lines, active Ras and its downstream signaling were inhibited by FTS [19, 22].

### Combined treatment with FTS + MPA inhibits USPC1 cell proliferation

**FIGURE 2:**
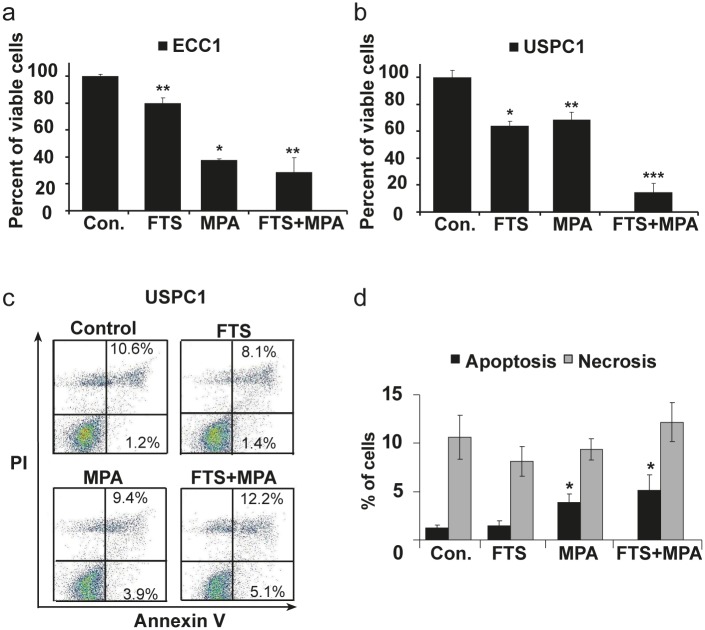
Effects of FTS and MPA on ECC1 and USPC1 cell viability (*a*) Combined treatment with FTS + MPA inhibits proliferation of ECC1 and USPC1 cells. ECC1 and USPC1 cells were plated in 24-well plates and treated after 24 hr with FTS (6 μM), MPA (10 nM), FTS (6 μM) + MPA (10 nM), or 0.1% DMSO (control). After 6 days cells were counted. (*a*) ECC1 cells showed the expected response of type 1 EC cells to MPA treatment, i.e., a decrease in their numbers that was significantly greater than the decrease obtained by treatment with FTS. The effect of treatment with FTS + MPA, again as expected, did not differ from the effect of MPA alone. (*b*) USPC1 shows the expected weak response of type 2 EC cells to MPA treatment. In combination with FTS, however, MPA caused a significant decrease in USPC1 cell proliferation. (*c* and *d*) MPA, but not FTS, induces apoptosis in USPC1 cells. For cell death evaluation, ECC1 and USPC1 cells were seeded in 6-well plates, treated for 5 days with FTS, MPA, or FTS + MPA (concentrations as above), and then collected and assayed by double staining with annexin-V-PI. Apoptosis and necrosis were determined respectively by staining with annexin-V or by double-staining with annexin-V-PI (see Methods), and analysed by FACS. (*c*) Results for control, FTS-treated, MPA-treated and FTS + MPA-treated USPC1 cells are shown. Lower left: counts of live cells with no staining. Lower right: counts of apoptotic cells (stained with annexin V only). Upper right: counts of necrotic cells (double-stained with annexin V-PI). Upper left: counts of DMSO-treated (control) cells. (*d*) No significant difference in apoptosis is seen between cells treated with FTS alone and control cells. Compared to control, however, apoptosis was significantly increased in cells treated with MPA or with FTS + MPA. There were no significant differences in treatment-related effects on necrosis,. These results indicated that FTS does not induce apoptosis but rather inhibits cell proliferation, whereas MPA induces apoptotic cell death. Results are presented as means ± SEM, *n* = 6. *, ** and *** are compared with the control for each cell line. *p < 0.05, ** *p* < 0.01, ***p < 0.001. Con, control; FTS, S-farnesylthiosalicylic acid; MPA, medroxyprogesterone acetate; PI, phosphatidylinositide.

We examined the effects of FTS, MPA, and FTS +MPA on the proliferation of ECC1 and USPC1 cells (Figs. 2*a* and 2*b*). Results were calculated as percentages of control. Number of ECC1 cells were reduced to 80.1 ± 3.8% by treatment with FTS (*n* = 6, *p* < 0.001), to 37.8 ± 0.9% by treatment with MPA (*n* = 6, *p* < 0.001), and to 28.6 ± 10.5% by the combined treatment (*n* = 6, *p* < 0.001). The numbers of USPC1 cells were reduced to 63.9 ± 3.6% by FTS (*n* = 6, *p* = 0.04), to 68.4 ± 5.8% (*n* = 6, *p* = 0.04) by MPA, and to 14.2 ± 6.9% by their combination (*n* = 6, *p* < 0.001). The finding that ECC1 cells were affected by MPA alone was expected, as these well-differentiated cells express active PRs and ERs [33]. The poorly differentiated USPC1 cells responded weakly to MPA alone, but were strongly affected by the combined treatment with MPA and FTS (Fig. 2*b*). These results indicated that FTS increases the sensitivity of USPC1 cells to MPA.

### MPA but not FTS induces apoptosis in USPC1 cells

Next we examined the nature of the reductions in ECC1 and USPC1 cell numbers observed after treatment with MPA, FTS, or their combination. To investigate the possible role of apoptosis or necrosis or both, we stained the cells with annexin-V (which stains apoptotic cells) or double-stained them with PI and annexin (which stains necrotic cells) (see Methods), and then analyzed the stained cells by FACS cytometry. Figure 2*c* presents the results obtained for the USPC1 cells; the results obtained for the ECC1 cells were similar (not shown). Data obtained for the control and after treatments with FTS, MPA, and FTS + MPA are shown as indicated in the four panels of Figure 2*c*. The lower left part of each square shows the counts of live cells with no staining; the lower right part shows the counts of apoptotic cells (stained with annexin V only); and the upper right part shows the counts of the double-stained necrotic cells. Relative to the control cells, there was no significant increase in apoptosis after treatment with FTS alone (apoptotic cells in the control amounted to 1.2 ± 0.3% of the total number (*n* = 6), and 1.4 ± 0.5% of the total number in the FTS-treated cells (*p* = 0.37, *n* = 6). Treatment with MPA resulted in a significant increase in apoptosis compared to that in the control (3.9 ± 0.8% of total MPA-treated cells; *p* = 0.01, *n* = 6), while in cells treated with the FTS + MPA combination 5.1 ± 1.6% of the total number were apoptotic (*p* = 0.02, *n* = 6). There were no significant differences in the numbers of necrotic cells seen after the different treatments (see Fig. 2*d*). These results indicated that FTS does not induce apoptosis but reduce cell numbers by inhibiting Ras and its signaling, which in turn inhibited proliferation (Fig. 1), whereas MPA reduced cell numbers via induction of apoptotic cell death (Fig. 2*b*).

### ERα and its phosphorylation at Ser118 and at Ser167 are downregulated by FTS in ECC1 and USPC1 cells

Phosphorylation of ERs activates them to regulate gene expression [12]. We examined two serine residues that are the main phosphorylation sites located within the activation function 1 region of the N-terminal domain of ERα [34]. These phosphorylations result in enhanced ER-mediated transcription [34]. Activation by phosphorylation of the MAPK/ERK pathway enhances phosphorylation of ERα Ser118. Activation by phosphorylation of the Akt pathway leads to phosphorylation of ERαSer167 [34]. Both of these pathways are regulated by Ras.

**FIGURE 3:**
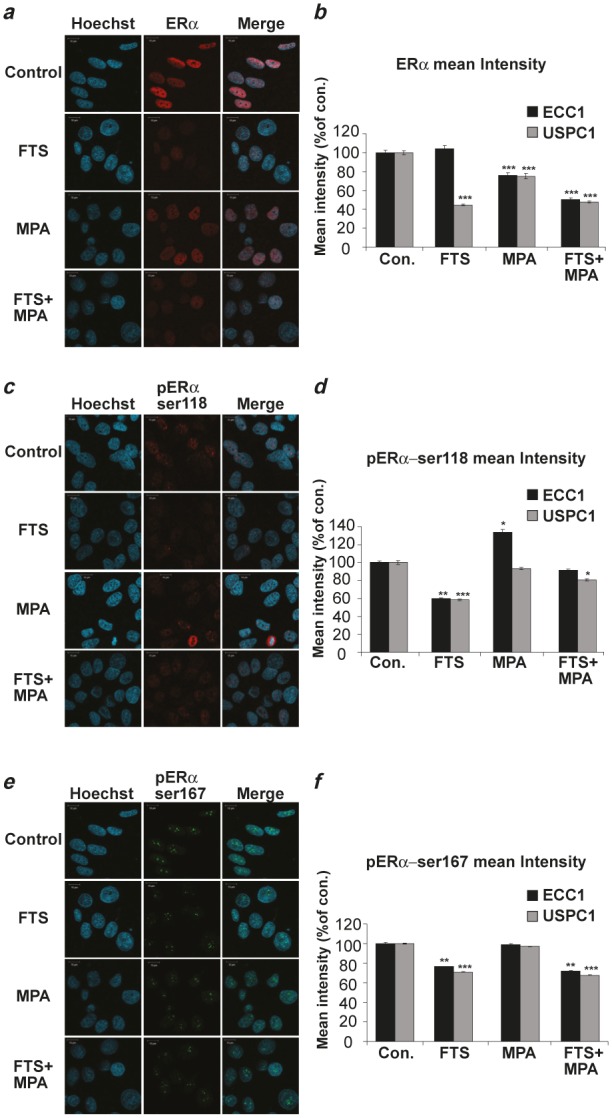
ERα and its phosphorylated forms are downregulated by FTS in ECC1 and USPC1 cell lines ECC1 and USPC1 cells were plated on glass cover slips in 6-well plates. After 24 hr the media were replaced with 0% FCS media (starvation). After 16 hr the cells were treated with FTS, MPA, FTS + MPA (concentrations as in Fig. 2), or 0.1% DMSO (control). Three hours after treatment the cells were stained with anti-ERα, anti-pERαSer118 or anti-pERαSer167 antibodies in an immunofluorescence experiment and subjected to confocal microscopy. Typical images of USPC1 cells stained with (*a*) anti-ERα antibody, (*c*) anti-pERα Ser118 antibody, and (*e*) anti-pERα Ser167 antibody are shown. Hoechst stain (blue) was used for nuclear staining. Statistical analysis of mean fluorescence intensity of (*b*) ERα (*d*) pERαSer118, and (*f*) pERαSer167 are presented as percentages of control (DMSO-treated) ECC1 and USPC1 cells (means ± SEM, *n* = 100). ERα was located mainly in the nucleus, not in the cytoplasm. FTS significantly reduced phosphorylation of Ser118 and of Ser167, while MPA caused a decrease in total ERα in USPC1 cells but not in the phosphorylated forms. These findings correlated with the results showing that FTS, but not MPA, downregulates pathways of active Ras signaling. *, ** and *** are compared with the control for each cell line. *p < 0.05, ** *p* < 0.01, ***p < 0.001. Con, control; ERα, estrogen receptor alpha; FTS, S-farnesylthiosalicylic acid; MPA, medroxyprogesterone acetate; Ser, serine.

To determine the amount and localization of ERα and its phosphorylated forms we treated ECC1 and USPC1 cells with FTS, MPA, FTS + MPA, or 0.1% DMSO (control) and assayed them in immunofluorescence experiments using anti-ERα, anti-pERα Ser118, and anti-pERα Ser167 antibodies. Hoechst staining was used to check nuclear localization. Figures 3*a*, 3*c* and 3*e* show typical results of staining of USPC1 cells with ERα, pERα Ser118 and pERα Ser167, respectively, for each of the four treatments. The results confirmed that the receptor, as already known [35], is localized mainly in the nucleus. Statistical analyses of the mean fluorescence intensities of ERα, pERα Ser118 and pERα Ser167, expressed as percentages of control in ECC1 and in USPC1 cells, are presented in Figures 3*b*, 3*d* and 3*f*, respectively. ERα in ECC1 cells showed no change after FTS treatment (*p* = 0.24), but was strongly downregulated by FTS in USPC1 cells (44.8 ± 0.23%, *p* << 0.0001, *n* = 100). Treatment with MPA caused a decrease in ERα in both cell lines (ECC1: 76.2 ± 2.5%, *p* << 0.0001, *n* = 100; USPC1: 75.2 ± 2.8%, *p* << 0.0001, *n* = 100). ERα was also reduced in both cell lines by treatment with the FTS + MPA combination (ECC1: 50.4 ± 1.5%, *p* << 0.0001, *n* = 100; USPC: 47.6 ± 1.2%, *p* << 0.0001, *n* = 100).

Next we examined the intensities of pERα Ser118 and pERα Ser167 in ECC1 and USPC1 cells under the different treatments. pERα Ser118 and pERα Ser167 both showed nuclear labeling by their relevant antibodies (Figs. 3*c* and 3*e*). FTS, but not MPA, strongly reduced pERα Ser118 phosphorylation in both ECC1 (60.1 ± 0.6%, *p* << 0.0001, *n* = 100) and USPC1 cells (58.4 ± 0.7%, *p* << 0.0001, *n* = 100, Fig. 3d). The combined treatment did not improve the FTS effects (91.3 ± 1.3%, *p* = 0.1, *n* = 100 in ECC1; 80.6 ± 1.1%, *p* = 0.01, *n* = 100 in USPC1 cells). Similar results were obtained for pERαSer167: FTS treatment reduced pERαSer167 in both ECC1 (76.6 ± 0.4%, *p* << 0.0001, *n* = 100) and USPC1 cells (70.8 ± 0.2%, *p* << 0.0001, *n* = 100), MPA had no effect (*p* = 0.45, *n* = 100 in ECC1; *p* = 0.29, *n* = 100 in USPC1 cells), and FTS + MPA reduced it to 71.9 ± 0.6% (*p* << 0.0001, *n* = 100) in ECC1 cells and to 67.7 ± 0.2% (*p* << 0.0001, *n* = 100) in USPC1 cells. Altogether, these results suggested that FTS, but not MPA, inhibits ERα phosphorylation in both ECC1 and USPC1 cells. This finding correlates with the observed FTS-induced reduction in pERK and pAkt (Fig. 1*b*), and supports the conclusion that once the phosphorylation levels are decreased, activation of ERα is also decreased [12].

### MPA increases ERα degradation in ECC1 and in USPC1 cells

**FIGURE 4:**
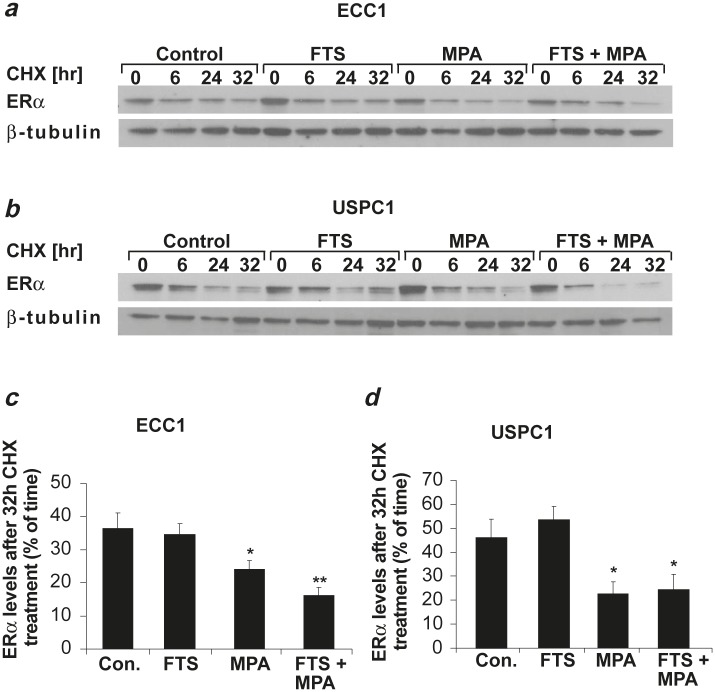
MPA increases ERα degradation in ECC1 and USPC1 cell lines ECC1 and USPC1 cells were plated in 6-well plates, and after 24 hr they were treated with FTS, MPA, FTS + MPA (concentrations as in Fig. 2), or 0.1% DMSO (control) (four wells per treatment). After 5 days, one well from each treatment was lysed (time zero) and the other three wells were treated with CHX (50 μg/ml). At 6, 24, or 32 hr after CHX addition the cells were lysed and subjected to western blot analysis with anti-ERα antibody. β-tubulin was used as loading control. Results of a typical experiment are presented, showing ERα and β-tubulin expression levels (*a*) in ECC1 cells and (*b*) in USPC1 cells. The blot was loaded with samples of control, FTS-treated, MPA-treated, and FTS + MPA-treated cells taken at time zero and at 6, 24 and 32 hr after addition of CHX. (*c*) Statistical analysis of ERα after treatment with CHX for 32 hr, presented as percentages of ERα at time zero, for each of the treatments of ECC1 cells and (*d*) of USPC1 cells. ERα stability in both cell lines was significantly reduced after treatment with MPA and with FTS + MPA, suggesting that ERα degradation is increased by MPA but not by FTS. Results are shown as means ± SEM (*n* = 4). * and ** are compared with ERα levels, 32 hr after CHX addition, expressed as a percentage of the control at time zero in each cell line. *p < 0.05, ** *p* < 0.01. CHX, cycloheximide; Con, control; ERα, estrogen receptor alpha; FTS, S-farnesylthiosalicylic acid; MPA, medroxyprogesterone acetate.

Next we examined whether the decrease in ERα was related to an increase in the receptor’s degradation. We treated ECC1 and USPC1 cells for 5 days with DMSO (control), FTS, MPA or FTS + MPA, and then treated them with CHX (50 μg/ml) to inhibit protein synthesis. At 0, 6, 24 and 32 hr after addition of CHX the cells were lysed for western blot analysis with anti-ERα antibody. Figures 4*a* and 4*b* depict ERα expression levels in ECC1 and USPC1 cells, respectively, following each of the treatments, at time zero and at 6, 24, and 32 hr after CHX addition (β-tubulin was used as a loading control). A reduction in receptor levels was already observed by 6 hr after CHX addition and increased with time. This finding led us to choose 32 hr post CHX treatment as the time point for comparison of ERα expression levels in the different treatments. Figures 4*c* and 4*d* show the relative expression levels of ERα in ECC1 and USPC1 cells, respectively, at 32 hr, presented as a percentage of the expression at time zero, for each of the treatments. In the absence of drugs (control), ERα was reduced to 36.3 ± 4.7% (*p* < 0.001, *n* = 4) in ECC1 and to 46.3 ± 7.6% (*p* < 0.001, *n* = 4) in USPC1 cells. After FTS treatment the receptor was reduced to 34.6 ± 2.2% (*p* < 0.001, *n* = 4) in ECC1 and to 53.4 ± 5.4% (*p* < 0.001, *n* = 4) in USPC1 cells. MPA treatment reduced the receptor to 24.0 ± 2.7% (*p* < 0.001, *n* = 4) in ECC1 and to 22.4 ± 5.1% (*p* < 0.001, *n* = 4) in USPC1 cells. Treatment with the FTS + MPA combination reduced ERα to 16.1 ± 2.6% (*p* < 0.001, *n* = 4) in ECC1 and to 24.5 ± 6.4% (*p* < 0.001, *n* = 4) in USPC1 cells. At 32 hr post CHX addition, comparison of expression levels of ERα for the different treatments with its expression level in the control showed that there was no significant difference in ERα degradation with FTS treatment in ECC1 cells (*p* = 0.386, *n* = 4) or in USPC1 cells (*p* = 0.238, *n* = 4), whereas MPA increased the receptor’s degradation both in ECC1 (*p* = 0.03, *n* = 4) and in USPC1 cells (*p* = 0.02, *n* = 4). FTS + MPA also increased protein degradation in both ECC1 (*p* = 0.004, *n*= 4) and USPC1 cells (p = 0.03, n = 4). These results indicated that ERα degradation is enhanced by MPA but not by FTS.

Together with the findings (Fig. 2*d*) that MPA increases apoptotic cell death, these results show that the presence of ERα protein in EC cells protects them from apoptosis.

### Combined treatment with FTS + MPA decreases mRNA of ERα and increases mRNA of ERβ in USPC1 EC cells

**FIGURE 5:**
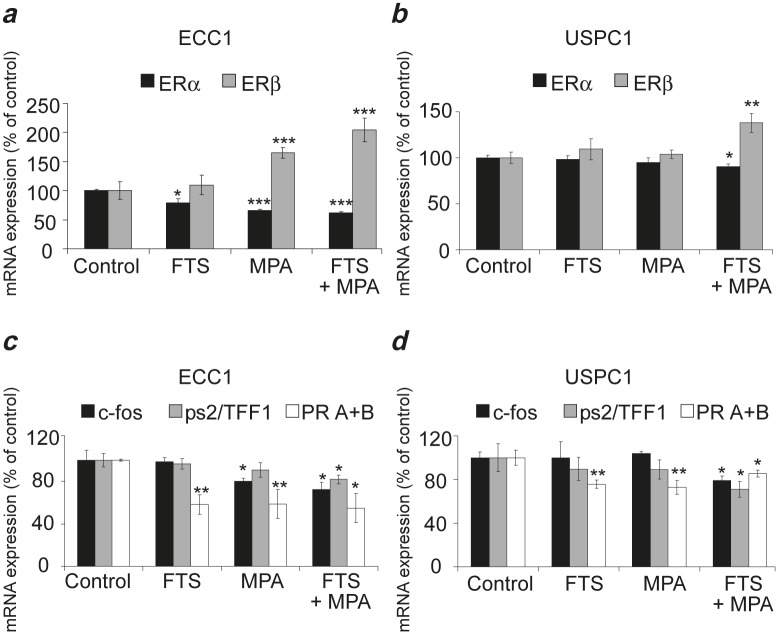
Combined treatment (FTS + MPA) decreases ERα mRNA and increases ERβ mRNA, leading to decreased ERα-mediated transcription in ECC1 and USPC1 cells ECC1 and USPC1 cells were plated in 10-cm plates and treated 24 hr later with FTS, MPA, FTS + MPA (concentrations as in Fig. 2) or 0.1% DMSO (control). After 6 days of treatment we assayed the mRNA of *ERα, ERβ*, *progesterone receptors A+B (PR A+B), ps2/TFF1* and *c-fos* in both cell types, using real-time PCR. *GAPDH* was used as a housekeeping gene. The mRNA level of each gene is presented as a percentage of its level in the control. (*a*) In ECC1 cells, ERα mRNA was reduced by treatment with FTS, MPA, or FTS + MPA. ERβ mRNA was increased by treatment with MPA and FTS + MPA. (*b*) In USPC1 cells, no change was induced by FTS or MPA treatment alone. With FTS + MPA, however, ERα mRNA was decreased and ERβ mRNA was increased. ERβ is known to modulate ERα transcriptional activity and its expression increases the degradation of ERα. Thus, these results showed that treatment with FTS + MPA causes USPC1 cells to respond to MPA by acting like ECC1 cells. (*c*) In ECC1 cells, PR A+B mRNA was decreased by all three treatments; ps2/TFF1 mRNA was decreased only by FTS + MPA, and c-fos mRNA was decreased by MPA and by FTS + MPA. (*d*) In USPC1 cells, a significant decrease in c-fos mRNA and ps2/TFF1 mRNA was observed only with FTS + MPA treatment. PR A+B mRNA in these cells was decreased by all three treatments, similar to the findings in ECC1 cells. The decrease in transcription of ERα-mediated genes as result of treatment with FTS + MPA showed that the combined treatment decreases the activity of ERα. Results are shown as means ± SEM (*n* = 6 for (*a*) and (*b*); *n* = 4 for (*c*) and (*d*)). *, **, and *** are compared with the relevant control. *p < 0.05, ** *p* < 0.01, *** *p* < 0.001. ERα, estrogen receptor alpha; ERβ, estrogen receptor beta; FTS, S-farnesylthiosalicylic acid; MPA, medroxyprogesterone acetate.

Next we wanted to find out whether the reduction in ERα expression by MPA + FTS is related to a decrease in ERα mRNA and whether ERβ plays a role in the mechanism of action of these drugs. ECC1 and USPC1 cells were plated in 10cm plates and treated after 24 hr with FTS, MPA, FTS + MPA (concentrations as above), or with 0.1% DMSO (control). After 6 days of treatment we assayed the mRNA of ERα and of ERβ in both cell lines using real-time PCR. The results for each treatment, shown in Figures 5*a* (ECC1) and 5*b* (USPC1), are expressed as percentages of the relevant control value (taken as 100%). In ECC1 cells treated with FTS, ERα mRNA was decreased to 78.8 ± 7.3% (*p* = 0.01, *n* = 6). In ECC1 cells treated with MPA, ERα mRNA was decreased to 66.0 ± 2.3% (*p* = 0.008, *n* = 6), and in ECC1 cells treated with FTS + MPA, ERα mRNA was decreased to 57.6 ± 0.9% (*p* < 0.0001, *n* = 6). ERβ mRNA in the FTS-treated ECC1 cells was unchanged (*p* = 0.4, *n* = 6). Treatment with MPA alone yielded a significant increase in ERβ mRNA (165.1 ± 9.2% (*p* = 0.002, *n* = 6), and treatment of these cells with FTS + MPA yielded an even larger increase of 204.4 ± 20.1% (*p* < 0.001, *n* = 6). In the USPC1 cells there was no significant difference either in ERα mRNA or in ERβ mRNA when the cells were treated with FTS alone and with MPA alone (*p* = 0.36 and *p* = 0.20, respectively, for ERα mRNA, *n* = 6; and *p* = 0.24, *p* = 0.30, respectively, for ERβ mRNA, *n* = 6). With the combined treatment (FTS + MPA), however, the difference was significant: ERα mRNA dropped to 90.2 ± 4.3% (*p* = 0.02, *n* = 6) whereas ERβ mRNA rose to 137.9 ± 10.2% (*p* = 0.004, *n* = 6).

These results can explain the difference in the action of MPA on type 1 and type 2 EC cells. In ECC1 cells (type 1 EC), MPA increased ERβ mRNA and decreased ERα mRNA. This phenomenon was enhanced when the cells were treated with FTS + MPA (Fig. 5*a*). These findings are consistent with the known role of ERβ as a regulator of ERα, which by itself causes cell proliferation [15, 16]. The results also reflect the known sensitivity of ECC1 cells to progestins. Downregulation of ERα transcription and upregulation of transcription of ERβ may explain the observed reduction in proliferation of FTS-treated and MPA-treated cells. In USPC1 cells (type 2 EC), which are not sensitive to progestins [2] (Fig. 5*b*), it was only the combined treatment of FTS + MPA that induced a decrease in mRNA of ERα and an increase in mRNA of ERβ. Thus, in the presence of FTS, the response of USPC1 cells to MPA was similar to that of MPA alone treated ECC1 cells (Figs. 5*a* and 5*b*). These results indicated that FTS converts type 2 EC cells to “type1-like” EC cells, which respond to progestins.

### Combined treatment with FTS and MPA decreases ERα-mediated gene transcription in ECC1 and USPC1 cells

Next we examined whether the FTS-induced decrease in ERα phosphorylation leads to downregulation of gene transcription. Studies have shown that transcription of the *PR A+B* [36], *ps2/TFF1* [37, 38], and *c-fos* [39-41] is induced by estrogen. *c-fos* is activated as a result of activation of the MAPK and PI3K signaling pathways. [13, 42] We therefore performed real-time PCR assays using primers for *PR, ps2/TFF1*, and *c-fos* genes. In ECC1 cells we found no significant differences in *ps2/TFF1* mRNA between cells treated with FTS alone and with MPA alone (*p* = 0.34, *p* = 0.18, *n* = 4, respectively), whereas a significant decrease (in all cases relative to control (taken as 100%) to 81.8 ± 4.1% was obtained with treatment by FTS + MPA (*p* = 0.02, *n* = 4) (Fig. 5*c*). Expression of *c-fos* mRNA was unchanged by treatment with FTS alone (*p* = 0.44, *n* = 4), but was reduced to 80.3 ± 2.9% with MPA alone (*p* = 0.04, *n* = 4) and to 72.5 ± 6.9% (*p* = 0.02, *n* = 4) with FTS + MPA (Fig. 5*c*). Expression of *PR A+B* mRNA was reduced in all treatment groups: it was reduced to 58.5 ± 9.2% (*p* = 0.002, *n* = 4) in the FTS-treated cells, to 58.8 ± 13.5% (*p* = 0.01, *n*=4) in the MPA-treated cells and to 54.8 ± 13.5% (*p* = 0.008, *n* = 4) in the FTS + MPA-treated cells (Fig. 5*c*). In USPC1 cells there were no significant differences in *ps2/TFF1* mRNA between the control and the cells treated with FTS alone or with MPA alone (*p* = 0.27, *p* = 0.25, *n* = 4, respectively) (Fig. 5*d*). The same was found with respect to *c-fos* mRNA (*p* = 0.38, *p* = 0.47, *n* = 4, respectively) (Fig. 5*d*). With the combined FTS + MPA treatment, however, significant differences were observed: *ps2/TFF1* mRNA decreased to 71.1 ± 8.3 (*p* = 0.04, *n* = 4) and *c-fos* mRNA decreased to 76.3 ± 4.6% (*p* = 0.04, *n* = 4). Expression of *PR* mRNA was reduced in all three treatment groups: to 75.7 ± 3.9% with FTS (*p* = 0.006, *n* = 4), to 72.8 ± 6.5% with MPA (*p* = 0.008, *n* = 4), and to 85.3 ± 3.2% with FTS + MPA (*p* = 0.04, *n* = 4) (Fig. 5*d*). The observed differences in mRNA expression between the genes point to the likelihood of different mechanisms driving their transcription. The reduction in mRNA expression of *c-fos* correlates with the observed reduction in active Ras pathways by FTS (Fig. 1*b*). The reduced transcription of three ER-regulated genes in both ECC1 and USPC1 cells observed with FTS + MPA strongly suggested that the combined treatment reduces not only the mRNA and protein expression of ERα but also ERα activity.

## DISCUSSION

Progestin therapy is known to be an effective treatment for patients with well-differentiated EC [8, 9]. It offers an optional treatment for poor surgical candidates and for patients who wish to preserve fertility. Progestins antagonize ERα, leading to inhibition of tumor growth. Treatment efficacy declines with the severity and the de-differentiation stage of the tumor [1]. Therefore, increasing research efforts are being made to sensitize high-grade EC to progestins. The beneficial effects of combined treatment with different drugs designed to treat EC can be enhanced by targeting different pathways of oncogenic signaling. One such approach is based on the combination of progestins with chemotherapy or with molecular-targeted therapies [43]. For example, combinations of MPA and 5-aza-2’-deoxycytidine (5-aza-CdR) [44] or metformin [45] were found to be effective against EC cells by enhancing the anti-proliferative effect of MPA. Moreover, gonadotropin-releasing hormone (GnRH) agonists (such as leuprolide acetate) in combination with Mirena, a levonorgestrel-releasing intrauterine delivery system, were shown to be effective in clinical studies [46, 47].

**FIGURE 6:**
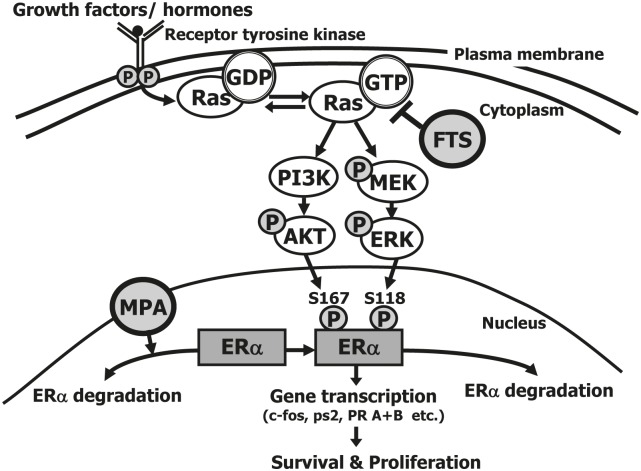
Proposed mechanism of the combined action of FTS + MPA on EC cells Estrogens play an important role in the regulation of cell proliferation, differentiation, and function of the endometrium. They mediate their biological effects through two estrogen receptors in the nucleus, namely ERα and ERβ. ERs are transcription factors that are activated by phosphorylation and control gene expression. The activation is induced either in response to ligand binding or independently of ligand. Ras becomes active in the Ras-GTP form, which is upregulated by extracellular signals such as growth factors and hormones that bind the tyrosine kinase receptor. Once activated, Ras-GTP signals to multiple effector pathways that regulate proliferation, survival, metabolism, migration and shape of the cell. One of these pathways causes phosphorylation of Akt, which leads to cell survival, and another causes phosphorylation of ERK, leading to cell proliferation. Nuclear ERα is phosphorylated by pAkt at Ser167 and by pERK at Ser-118. Once phosphorylated, ERα is activated to target the transcription of genes (such as *c-fos, ps2/TFF1*, and *PR A+B*), leading to cell survival and proliferation. MPA increases ERα degradation, thereby reducing ERα in the cell (both in the nucleus and in the cytoplasm). FTS inhibits active Ras-GTP, leading to a decrease in pAkt and pERK, and hence a decrease in pERαSer118 and in pERαSer167, and finally a decrease in ERα gene transcription. These results showed that the combination of MPA + FTS inhibits proliferation of endometrial cells. ERα, estrogen receptor alpha; FTS, S-farnesylthiosalicylic acid; MPA, medroxyprogesterone acetate; PI3K, phosphatidylinositide 3-kinase; s118, serine-118; s167, serine-167.

In this study we examined the effect of treatment with a combination of the Ras inhibitor FTS and the progestin MPA on proliferation and death of both the well-differentiated ECC1 type 1 EC cells and the poorly differentiated USPC1 type 2 EC cells. Our results show that FTS increased the antiproliferative effects of MPA on type 2 EC, which otherwise responds poorly to MPA (Fig. 2*b*). Figure 6 summarizes the proposed mechanism of the combined FTS + MPA treatment of EC cells. MPA induces degradation of ERα in both ECC1 and USPC1 cells, as observed in this study also with the combined drug treatment (Fig. 4 and Fig. 6). Thus, treatment with this progestin leads to a reduction in total ERα in the nucleus of USPC1 cells (Figs. 3*a* and 3*b*). MPA also caused apoptotic cell death (Fig. 2*b*). Taken together, these results suggest that ERα protects EC cells from apoptosis, as also shown previously in EC cells [48], in breast cancer cells [49] as well as pancreatic beta cells [50]. FTS inhibits proliferation of ECC1 and USPC1 cells (Fig. 1*a*) by downregulating active Ras-GTP and its downstream signaling pathways (Fig. 1*b* and Fig. 6). These signaling pathways are critical for ERα phosphorylation, which leads to ligand-independent gene transcription [12, 34]. These genes play critical roles in cell proliferation and death [13]. Two ERα sites that are phosphorylated by these pathways are Ser118 (activated by pERK) and Ser167 (activated by pAkt) [34]. We showed that Ras inhibition decreases phosphorylation of ERα in the nucleus (Fig. 3 and Fig. 6). In addition, combined treatment with FTS + MPA decreases the transcription of important ERα-regulated genes both in ECC1 and in USPC1 cells (Figs. 5*c*, 5*d* and Fig. 6), manifested by a decrease in ERα mRNA expression and an increase in mRNA expression of ERβ, known to inhibit ERα and cell growth [12] (Fig. 5*a* and 5*b*). Thus, treatment of USPC1 type 2 EC cells with FTS + MPA leads to lower ERα mRNA and higher ERβ mRNA expression, as seen in type 1 EC cells that respond well to MPA.

There are several cellular mechanisms of ER activation that lead to gene transcription. In the classical mechanism ER is activated by ligand binding, and in the nonclassical mechanism by ERK and Akt phosphorylations [12]. MPA reduced ligand-dependent ER activity, thereby antagonizing ERα and increasing its degradation, while FTS reduced ligand-independent ER activity by reducing ERα phosphorylation. It was only once these two ER-activation mechanisms were downregulated that we observed a decrease in the transcription of ER-mediated genes in USPC1 cells (Fig. 6).

We conclude that MPA antagonizes the unphosphorylated ERα. Once ERα phosphorylation is reduced by FTS, the EC cells become more sensitive to the antiproliferative effect of MPA. This is reminiscent of the observed enhanced sensitivity of EC cells to MPA when the latter is combined with the DNA demethylating agent 5-aza-CdR [44]. However unlike 5-aza-CdR [51], FTS is a non-toxic drug. Here we show that when the phosphorylation of ERα is decreased and ERβ mRNA is increased by FTS, USPC1 cells respond better to MPA.

In conclusion, MPA in combination with FTS leads to enhanced apoptotic death of EC cells. This can be used as new method to inhibit type 2 endometrial carcinoma.

## Material and methods

### Cell culture and reagents

The endometrial carcinoma type 1 (ECC1) cell line was provided by Dr. Haim Werner, Human Genetics Faculty of Medicine, Tel-Aviv University. The uterine serous papillary carcinoma-1 type 2 (USPC1) cell line was provided by Dr. Santin Alessandro, Yale University and sequenced by him [30]. ECC1 and USPC1 cells were maintained in DMEM and RPMI-1640 medium, respectively (Biological Industries, Kibbutz Beit Ha-Emek, Israel) supplemented with 10% fetal calf serum (FCS) (or with 5% FCS for experiments), 100 units/ml penicillin, 100 μg/ml streptomycin, 0.05 mg/ml gentamicin sulphate, and 25 ng/ml amphotericin B. All reagents were purchased from Biological Industries.

Cells were incubated at 37^°^C in a humidified atmosphere of 95% air / 5% CO_2_. FTS was kindly donated by Concordia Pharmaceuticals (Fort Lauderdale, FL). Cycloheximide (CHX) and MPA were purchased from Sigma-Aldrich (Rehovot, Israel).

### Proliferation and cell survival assay

For the FTS dose-response experiment we plated ECC1 cells (1.5 × 10^3^ cells per well) and USPC1 cells (4.5 × 10^3^ cells per well) in 24-well plates. After 24 h the cells were treated with FTS at different concentrations (in μM: 100, 75, 50, 25, 12, 6) or, as a control, with 0.1% dimethyl sulfoxide (DMSO). Cells were counted after 4 days.

To measure cell survival after treatment with FTS, MPA, or their combination (FTS + MPA), we plated ECC1 and USPC1 cells in 24-well plates as described above. After 24 h, the cells were treated with FTS (6 μM), MPA (10 nM), FTS + MPA (6 and 10 nM, respectively), or 0.1% DMSO (control). After 6 days cells were counted.

### Immunoblot analysis

ECC1 and USPC1 cells were each plated at a density of 5×10^5^ cells per 10-cm plate, and treated 24 h later with FTS (50 μM) or 0.1% DMSO (control). After 3 days cells were lysed as described [31]. The lysates (75 μg protein) were immunoblotted with mouse anti-pan-Ras Ab (1:2,500, Calbiochem, San Diego, CA), rabbit anti-Akt Ab (1:1,000), rabbit anti-phospho-Akt (anti-pAkt) Ab (1:1,000), mouse anti-phospho-ERK (anti-pERK) Ab (1:10,000, Sigma-Aldrich), rabbit anti-ERK Ab (1:1,000) and, as a loading control, rabbit anti-β-tubulin Ab (1:500, Santa Cruz Biotechnology, Santa Cruz, CA). Ras-GTP was assayed in lysates by GST-RBD pull-down assay as described previously [31]. Immunoblots were exposed to the appropriate secondary peroxidase-coupled IgG (1:2,500, Jackson ImmunoResearch Laboratories, West Grove, PA) and subjected to enhanced chemiluminescence (Amersham Pharmacia Biotech, Piscataway, NJ). Protein bands were quantified by densitometry with Image EZQuant-Gel statistical analysis software.

### Cyclohexamide assay for translation arrest

ECC1 and USPC1 cells were each plated in 6-well plates at a density of 2×10^5^ cells per well. After 24 hours the cells were treated with FTS, MPA, FTS + MPA or, as a control, DMSO (concentrations as above; 4 wells for each treatment). After 5 days one well from each treatment was lysed (time zero) and the rest of the wells were treated with CHX (50 μg/ml). At 6, 24, and 32 hr after CHX addition, cells were lysed for western blot analysis with mouse anti-ERα Ab (1:1000; Santa Cruz Biotechnology). Rabbit anti-β-tubulin Ab (1:1000) served as a loading control. Each experiment was carried out in duplicate and performed four times. Protein bands were quantified as described above.

### Fluorescence-activated cell-sorter (FACS) analysis

To quantify cell death, we seeded ECC1 and USPC1 cells in 6-well plates at a density of 2×10^5^ cells per well and treated them 24 h later with FTS, MPA, FTS + MPA, or, as a control, DMSO, at the above mentioned concentrations. After 5 days the cells were collected and assayed by double staining with annexin-V-phosphatidylinositide (PI) (IQ Products, Groningen, The Netherlands) according to the manufacturer’s instructions. The results obtained by FACSCalibur flow cytometry were analyzed with FlowJo Software (Ashland, OR). All experiments were carried out in duplicate and performed three times.

### Total RNA purification and real-time PCR analysis

ECC1 and USPC1 cells were each plated at a density of 5×10^5^ cells per 10-cm plate and treated 24 hours later with FTS, MPA, FTS + MPA or, as a control, DMSO (concentrations as above). After 6 days of treatment total RNA was isolated from the cells using the PerfectPure RNA Cultured Cell Kit (5 PRIME). Purified RNA was subjected to real-time PCR as described [32]. The primers used to target the *ER*α gene were 5’-CCACCAACCAGTGCACCATT-3’ (forward) and 5’-GGTCTTTTCGTATCCCACCTTTC-3’’(reverse); to target the *ER*β gene we used 5’-AGAGTCCCTGGTGTGAAGCAAG-3’ (forward) and 5’-GACAGCGCAGAAGTGCATC-3’ (reverse). To target the *PR A+B* genes we used 5’-CGCGCTCTACCCTGCACTC-3’ (forward) and 5’-TGAATCCGGCCTCAGGTAGTT-3’ (reverse); to target *c-fos* we used 5’-GGGGCAAGGTGGAACAGTTAT-3’ (forward) and 5’-CCGCTTGGAGTGTATCAGTCA-3’ (reverse); and to target *ps2/TFF-1* we used 5’-AGGCCCAGACAGAGACGTGTAC-3’ (forward) and 5’-CGTCGAAACAGCAGCCCTTA-3’ (reverse). To target the housekeeping gene *GAPDH* we used 5’-CCAGAACATCATCCCTGC-3’ (forward) and 5’-GGAAGGCCATGCCAGTGAGC-3’ (reverse). The mRNA expression of each target gene was normalized to the expression of *GAPDH* as a reference gene. Results were analyzed using Sequence Detection Software. All experiments were carried out in duplicate and performed three times.

### Immunofluorescence and confocal microscopy

ECC1 and USPC1 cells were plated on glass cover slips in 6-well plates at the densities mentioned above. After 24 hours the media were replaced by medium without FCS (starvation). After 16 h the cells were treated with FTS, MPA, FTS + MPA or, as a control, DMSO (concentrations as above) for 3 h and then stained with rabbit anti-pERα Ser167 (D1A3) Ab, mouse anti-pERα Ser118 (16J4) Ab (1:50; Cell Signaling, Danvers, MA), and mouse anti-ERα Ab (1:200; Santa Cruz Biotechnology), as described [32].

Slides were examined at a magnification of 60x with an LSM 510 META fluorescence microscope. Data were analyzed by Image Processing and Analysis in Java Software (ImageJ). Statistical analysis was carried out on 100 cells from each treatment. For both cell lines, experiments with each antibody performed three times.

### Statistical analysis

Data are expressed as means ± SEM. Significant differences between mean values were assessed by Student’s *t*-test. Values of *p* ≤ 0.05 were considered significant.
